# Barriers and facilitators to the integration of cervical cancer screening and treatment service with antiretroviral therapy follow-up clinics for women living with HIV in Northwest Ethiopia: a qualitative study

**DOI:** 10.3389/fpubh.2025.1552524

**Published:** 2025-06-19

**Authors:** Kelemework Gashinet Ferede, Addisu Gasheneit Ferede, Asiya Mohammed Abdu, Birhaneslasie Gebeyehu Yazew, Zewdu Bishaw Aynalem, Eneyew Talie Fenta, Daniel Shitu Mekonnen, Berihun Bantie, Gebiyaw Wudie Tsegaye

**Affiliations:** ^1^Department of Epidemiology and Biostatistics, College of Medicine and Health Sciences, Bahir Dar University, Bahir Dar, Ethiopia; ^2^Institute of Public Health, College of Medicine and Health Sciences, University of Gondar, Gondar, Ethiopia; ^3^Department of Nursing, College of Medicine and Health Sciences, Injibara University, Injibara, Ethiopia; ^4^Department of Public Health, College of Medicine and Health Sciences, Injibara University, Injibara, Ethiopia; ^5^Department of Comprehensive Nursing, College of Health Science, Debre Tabor University, Debre Tabor, Ethiopia

**Keywords:** Awi, cervical cancer, HIV, integration, screening, Ethiopia

## Abstract

**Background:**

Women living with HIV (WLHIV) face a significantly elevated risk of invasive cervical cancer, an AIDS-defining malignancy. Integrated cervical cancer screening (CCS) and HIV care are essential for optimal management; however, these services remain largely unintegrated in Ethiopia, resulting in low screening coverage. This study aimed to explore the barriers and facilitators to integrating CCS and preventive treatment services within antiretroviral therapy (ART) follow-up clinics for WLHIV in Northwest Ethiopia.

**Methods:**

A phenomenological study was conducted from January to April 2023 among 33 participants across nine purposively selected health facilities in Northwest Ethiopia. Data were collected using focus group discussions and in-depth key informant interviews. Audio-recorded data were transcribed, translated, and coded, followed by thematic analysis using Atlas. Ti 7 software.

**Results:**

Key barriers to integration included inadequate facilities, a shortage of dually trained clinicians, low healthcare provider motivation, the absence of integration policies, and a lack of evidence on integration effectiveness. Facilitators included the accessibility of free ART and CCS services, the simplicity of the visual inspection with acetic acid (VIA) screening method, the availability of staff trained in either ART or CCS, and patient familiarity with ART providers.

**Conclusion:**

Integrating cervical cancer screening with HIV care in Northwest Ethiopia encounters substantial obstacles, including facility limitations, clinician training deficits, motivational issues, policy gaps, and a lack of evidence. However, accessible services, the VIA method’s simplicity, and existing staff training provide opportunities for successful integration. Addressing these barriers and leveraging facilitators is crucial to improve integrated service delivery and reduce related mortality among WLHIV.

## Introduction

Cervical cancer (CC) is a gynecological malignancy arising from uncontrolled cell mutations in the cervix and progresses through stages, with advanced stages indicating widespread metastasis (1–4). Globally, CC is the fourth most common cancer in women, with approximately 660,000 new cases and 350,000 deaths reported in 2022, predominantly (90%) in developing countries (5). As an AIDS-defining malignancy, CC disproportionately affects women living with HIV (WLHIV), particularly in high HIV-burden settings (6, 7). In 2018, 5.8% of WLHIV globally had CC, with 4.9% attributable to HIV infection (8). However, this proportion ranges from 5 to 40% in high HIV-burden regions (9). Notably, Sub-Saharan Africa (SSA) bears a significant burden, with 27.4 to 63.8% of global CC-HIV comorbidity, and over 20 cases per 100,000 women attributable to HIV (8–11). Consequently, WLHIV faces an elevated risk of invasive cervical cancer (ICC), six times higher than HIV-negative women, despite having greater disparities in high and low-income countries (7, 12–14). Furthermore, they experience more aggressive disease progression and poorer treatment outcomes (15–17). In Ethiopia, where the national CC screening (CCS) uptake remains low overall (14.79%) and among WLHIV (18.17%) (18, 19), the need for effective interventions is critical. The comorbidity of CC and HIV negatively impacts WLHIV’s quality of life, socioeconomic status, and mental health, increasing healthcare system burdens (20–25). Disparities in access to integrated sexual and reproductive health services and limited human papillomavirus (HPV) vaccination and screening in Ethiopia exacerbate the burden (18, 26). Early detection and treatment of precancerous lesions can significantly reduce the burden of CC (27). The available literature appreciates the benefits of integration, worrying about the challenges of integration, and preferences for integration. Integrating CCS and preventive treatment services with routine HIV care for WLHIV using either three models such as clinic-based training, service co-location, and coordinated care pathway (28, 29) has been identified as a promising strategy to improve access and outcomes by demonstrating the feasibility, acceptability, and cost-effectiveness (28, 30). However, lack of effective collaboration and coordination, skilled and incentivized health workers, supportive institutional structures and dedicated resources, and concerns and preferences regarding integration influenced integrated care (31, 32). As CC is preventable, the World Health Organization (WHO) launched a CC elimination initiative, a global strategy that aims for widespread vaccination, screening, and treatment, particularly targeting WLHIV aged 25–49 years for HPV DNA testing every 3 up to 5 years (27).

Despite national recommendations for CCS among WLHIV aged 15–49 years using visual inspection with acetic acid (VIA) (33, 34), these services often operate vertically and are not integrated with antiretroviral therapy (ART) follow-up clinics. This lack of integration represents a missed opportunity to address the high burden of CC in this vulnerable population.

To date, there is a lack of research exploring the specific barriers and facilitators to integrating CCS and preventive treatment services within anti-retroviral therapy (ART) follow-up clinics in Ethiopia. Understanding these factors is crucial for developing effective strategies to improve the uptake of these essential services for WLHIV. Therefore, this study aimed to explore the barriers and facilitators to integrating CCS and preventing treatment services with ART follow-up clinics for WLHIV in Ethiopia.

## Materials and methods

### Study design, setting, and period

A phenomenological study design was conducted in the Awi Zone, Northwest Ethiopia from January to April 2023. Participants (*n* = 33) were recruited from nine health facilities (HFs): Injibara General Hospital, Danglla Primary Hospital, Chagni Primary Hospital, Gimjabet Health Center, Tilili Health Center, Chagni Health Center, Addis Kidam Health Center, Injibara Health Center, and Danglla Health Center. Awi Zone provides healthcare services to a population of 1,342,324 through its 54 HFs (49 health centers and 6 hospitals, comprising 2 general and 4 district hospitals). Of the total population, 51% are female, and 22.5% are women of reproductive age. The HIV prevalence in the Awi Zone is 0.4%. During the data collection period, approximately 3,428 out of 4,087 HIV-positive women had been screened for CC. The general health service coverage in the Zone was reported as 72% in the 2022 Zonal Health Department annual report (35).

### Population and eligibility criteria

This study employed a purposive sampling technique to recruit participants from selected HFs. The target population comprised health facility leaders (HFLs) and healthcare providers (HCPs) directly involved in the provision of ART and CCS services. Eligible participants included nurses, public health officers, midwives specifically trained in CCS procedures, and physicians working within ART clinics and/or gynecology outpatient departments (OPDs). Participants who experienced severe illness during data collection and who had less than 6 months of employment at the facility or were recruited temporarily were excluded.

### Operational definitions

Integrated services: Integrated services refer to the co-location of CCS and HIV care within ART follow-up clinics, delivered by the same HCPs for WLHIV (29). Barriers: Barriers are factors that impede the delivery of integrated CCS and HIV care in ART clinics for WLHIV (31, 36). Facilitators: Variables or circumstances that enable the integration of CCS and HIV care (37).

### Study setting and participants’ selection

This qualitative study was conducted in nine HFs, purposefully selected from the 24 ART centers within the Awi Zone. These nine facilities were specifically chosen because they were the sole sites implementing integrated CCS and preventive treatment services alongside HIV care. This novel initiative was supported by the non-governmental organization: International Center for AIDS Care and Treatment Program (ICAP). This selection strategy ensured the study focused on sites with direct experience in implementing the integrated program, thereby maximizing the relevance and depth of the collected data. A purposive sampling strategy was employed to recruit participants who possessed valuable insights into the implementation process. Two distinct participant groups were targeted: the HCPs directly involved in service delivery and the HFLs responsible for program oversight.

**Figure 1:**
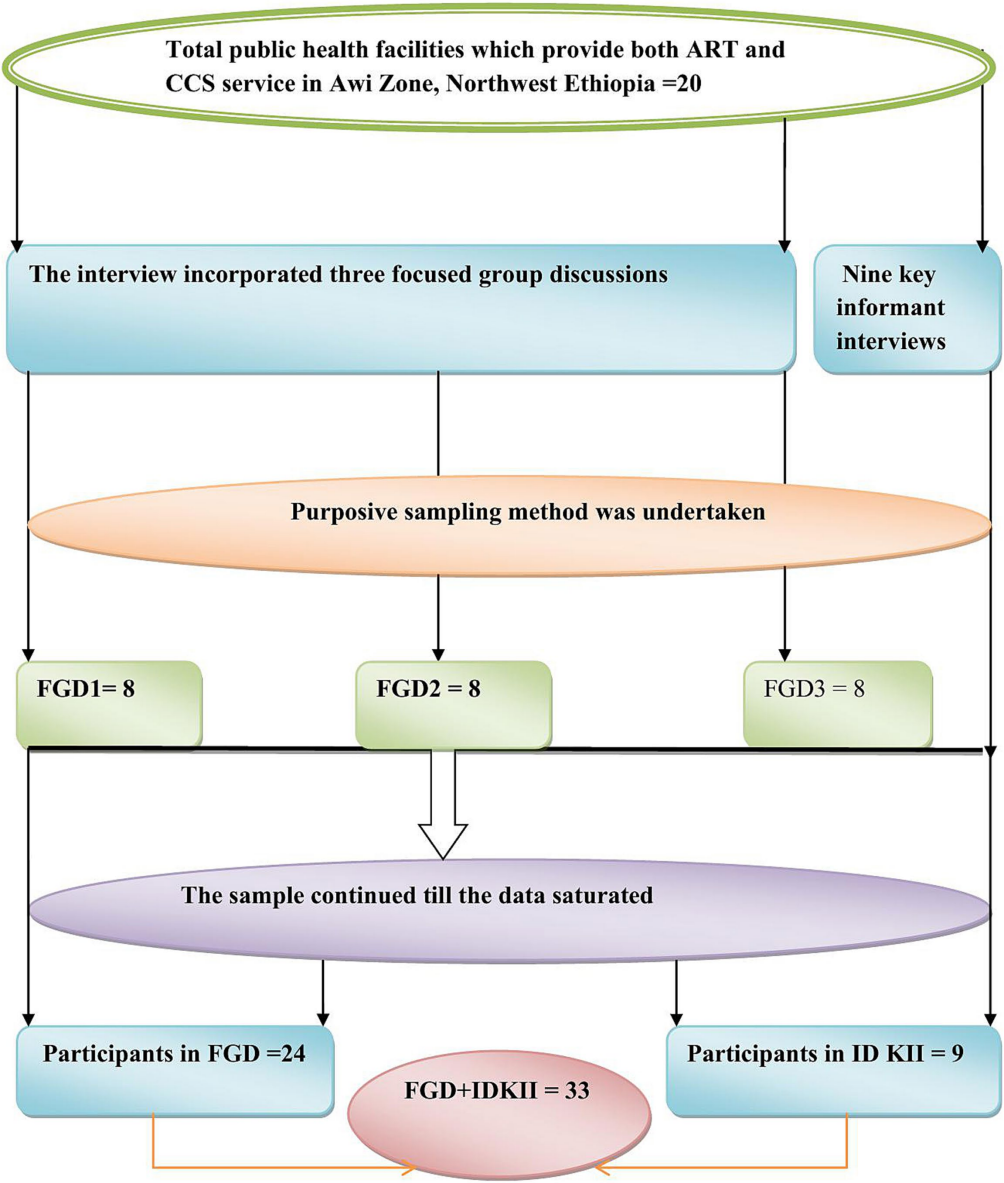
Schematic presentation of sampling procedure for barriers and facilitators to integration of CCS and treatment service with ART follow-up clinics, in Awi Zone, Northwest Ethiopia.

Three focus group discussions (FGDs), each comprising eight participants (totaling 24), were conducted with HCPs working in ART clinics and gynecological OPDs. Participants included nurses, midwives, health officers, and physicians. These professionals were selected based on their direct involvement in HIV care and their practical experience with the integrated CCS and preventive cryotherapy services, ensuring a comprehensive understanding of the operational challenges and successes. In total, nine in-depth key informant interviews (IDKIIs) were conducted with HFLs. These leaders were chosen for their detailed understanding of their respective facilities and their roles in managing the integrated service program. Their insights provided critical perspectives on the organizational and administrative aspects of implementation (Figure 1).

The sample size for both FGDs and IDKIIs was determined by the principle of information saturation. Data collection continued until no new themes or insights emerged, indicating that a comprehensive understanding of the research topic had been achieved. By employing a rigorous purposive sampling strategy and adhering to the principle of information saturation, this study ensured the collection of rich and diverse qualitative data, providing a nuanced understanding of the barriers and facilitators to integrating CCS and treatment services within ART follow-up clinics in Northwest Ethiopia.

### Data collection tools and procedures

The qualitative data for this study were gathered using a semi-structured interview guide, adapted from previous literature (31, 32, 38), carefully designed to elicit rich, nuanced data, including both verbal and non-verbal cues from participants. Developed initially in English, the interview guide underwent a rigorous translation and back-translation process by language experts to ensure semantic equivalence in Amharic, the language in which all discussions were conducted (Table 1). Prior to its use in the main study, the interview guide was pilot-tested with three participants to assess its clarity, relevance, and alignment with the study objectives. Data from the pre-test interviews were excluded from the final analysis.

**Table 1:** Interview guidelines were used to extract the data for barriers and facilitators to the integration of CC screening and HIV care for women living with HIV.

SN	Interview guidelines/questions
1	How do you understand the dual burden of Cervical cancer on women living with HIV, their families, communities, and one country?
2	Could you explain how early screening/detection for cervical cancer reduces the burden on women?
3	What are the challenges/barriers that hinder HIV-positive women from undergoing early screening for cervical cancer?
4	How do you see the integration of cervical cancer screening services into ART follow-up clinics, particularly when provided at a single unit by the same healthcare providers?
5	What is the importance of integrating the two services in terms of time, cost, and treatment outcome?
6	What are the enabling factors/facilitators of cervical cancer and HIV care service integration?
7	What are the barriers to delivering integrated CC screening services at ART Clinics? From different perspectives (Health system, H. care personnel, supplies, etc.)
8	What should be done to deliver and sustain the integrated CC screening and HIV care, to increase its accessibility for all HIV-positive women?

Eligible participants were invited by the principal investigator to partake in the discussions. To cultivate a safe and comfortable environment conducive to discussing potentially sensitive topics, a private setting was arranged for each interview. Before commencing, the principal investigator introduced himself, thoroughly outlined the study’s purpose and procedures, and obtained informed consent for audio recording. Participants were explicitly informed about the measures implemented to safeguard their emotional well-being throughout the discussion and were reminded of their right to withdraw from the study at any time without consequence.

A trained and experienced moderator, skilled in managing sensitive issues and participants’ emotional states, facilitated the discussions. The moderator employed active listening techniques, attentively responding to verbal and non-verbal cues. The pace of the discussions was carefully managed, with pauses and opportunities for breaks offered as needed to ensure participants’ comfort and well-being. Participants were consistently reminded of the voluntary nature of their participation and their freedom to take breaks or withdraw at any point. Furthermore, information regarding available support services related to HIV care was readily accessible to participants, although no instances of significant distress requiring these services were observed during the study.

To ensure the integrity of the audio data, participants were asked to silence their mobile phones. Anonymity was maintained through the assignment of a unique code to each participant, and basic demographic information was collected separately. The principal investigator guided the face-to-face interviews using flexible probing techniques to encourage comprehensive and detailed responses. All discussions were simultaneously captured using both a digital audio recorder and a smartphone as backups. A dedicated note-taker meticulously documented key points and participant codes during the discussions, ensuring a comprehensive record of the interactions. Rigorous attention was paid to note-taking accuracy, audio recording quality, and the natural flow of questions. Data collection continued until thematic saturation was achieved, indicated by the absence of new emerging themes or insights. In-depth interviews with HF leaders followed a similar protocol but were conducted on an individual basis. All audio recordings and field notes were securely stored on a password-protected computer in a secure location to guarantee confidentiality.

### Trustworthiness of the study

To ensure the rigor of this qualitative study, four established criteria were applied: credibility, confirmability, dependability, and transferability. Data triangulation was achieved through interviews with participants possessing diverse socio-demographic characteristics, complemented by audio recordings and detailed field notes. All participant information was meticulously documented to maintain a comprehensive audit trail. Prolonged engagement with the data allowed the investigators to develop a thorough understanding of the participants’ perspectives.

Member checking was employed by sharing data interpretations with selected participants, who were invited to validate or challenge the investigators’ understanding of their narratives and provide feedback on the findings. Peer debriefing, involving an external review by disinterested peers, was conducted prior to finalizing the results. To ensure confidentiality, participants’ names and other personal information were not recorded; instead, each participant was assigned a unique, temporary code.

### Data management and analysis

Qualitative data were analyzed using thematic analysis. Initially, researchers repeatedly listened to audio recordings of discussions and interviews to ensure data familiarization. Subsequently, audio-recorded data were transcribed verbatim into Amharic and then translated into English. Transcription accuracy was verified by all research team members through multiple reviews and cross-referencing with the original audio recordings. Following interpretation and meaning ascription, data were coded and categorized into main themes and subthemes using Atlas. Ti 7 qualitative data analysis software. Relevant verbatim quotes were selected to illustrate each theme and subtheme. Theme and subtheme development involved iterative reading of the transcripts. The categorized data were then repeatedly reviewed to draw conclusions based on both explicit and implicit meanings within the text. Finally, the findings were presented thematically.

### Ethical consideration

To ensure ethical conduct, the principal investigator explained the aims of the study and the necessity to participants before initiating interviews. Participants were also informed of their right to interrupt the interview at any time. Following a detailed explanation of the research purpose, verbal informed consent was obtained from each participant. To maintain confidentiality, participants were assigned unique identification codes, and they were instructed not to disclose their names. All names and other personal identifiers were removed throughout the study process and from the final report.

## Results

### Sociodemographic characteristics of participants in Awi zone, Northwest Ethiopia

The qualitative data were collected from 33 participants (24 in FGDs and 9 in IDKIIs) to explore barriers and facilitators to the integration of CCS services within ART follow-up clinics for WLHIV. A majority of participants were aged 30 and above. Approximately half were married, and the majority of participants were identified as Orthodox Christians. Over half (54.5%) of the HCPs and HFLs had 5–10 years of experience in their health professions (Table 2).

**Table 2:** Sociodemographic characteristics of the participants in FGDs and IDKII in Awi Zone, Northwest Ethiopia (*n* = 33).

Variables	Categories	Frequency (%)
Age in (years)	30 or younger	15 (45.5)
	Older than 30	18 (54.5)
Marital status	Single	8 (24.2)
	Married	20 (60.6)
	Divorced	5 (15.2)
Religion	Orthodox	22 (66.7)
	Muslim	7 (21.2)
	Protestants	4 (12.1)
Level of education	Diploma	5 (15.2)
	Degree	8 (24.2)
	Masters	14 (42.4)
	Medical Doctor	6 (18.2)
Work experiences (in years)	Less than five	5 (15.2)
	Five-Ten	18 (54.5)
	Greater than ten	10(30.3)
Type of profession	Physician	6 (18.2)
	Health officers (HOs)	8 (24.2)
	Nurses	10 (30.3)

### Barriers and facilitators to the integration of CCS and treatment services within HIV care in ART clinics

Analysis of participant responses revealed four primary themes influencing the integration of CCS and treatment services within ART clinics: accessibility-related factors, health institution-related factors, health system-related factors, and healthcare provider-related factors emerged as main themes. Each theme encompassed both barriers and facilitators to integrated service delivery. The following sections detail these themes, along with their respective subthemes and supporting codes.

### Theme-I: health system related barriers hindering efficient service integration

This theme highlights critical health system limitations that impede the effective integration of CCS services within ART follow-up clinics. These barriers directly impact the potential for streamlined service delivery and optimal resource utilization:

#### Sub-theme 1: policy vacuums obstructing integrated service delivery

The absence of clear national or regional policies for CCS and ART integration creates ambiguity, preventing standardized implementation and clear protocols despite the potential for shared resources and increased demand requiring additional supplies. A participant elaborated on these as:

*“As our institution, limited training program, absence of well-designed health policy, lack of materials such as cryogen, Koch / and, no apparent classroom for this activity [integration] are challenges of integrating the two [ART and CCS] services*.” (33 years, in FGD-3).

#### Sub-theme 2: unproven efficiency and outcomes deterring investment

The absence of strong data on the cost-effectiveness and clinical benefits of integrated CCS and ART services makes it difficult to secure resources and commitment for widespread implementation, despite potential efficiencies. Participants also voiced this concern:

*“In my point of view, regarding the service to be integrated, there is no evidence whether or not integration is effective, especially cost-effectiveness and patients` clinical outcome, furthermore, even if it is effective, no clear funding mechanisms for the implementation and sustainability of this service [integrated screening] for HIV positive women.” (28 years, in FGD-2).*

Likewise, another respondent also described the above idea as:

*“As it [integration] is (new initiative), especially for our institution, there are gaps in “funding mechanism,” there is no funding agency, we will start but it will terminate due to lack of funding agency, lack of research-based evidence on long term outcome of integration on both patient and provider perspectives.”* (36 years, IDKII-5).

Sub-theme 3: Resource scarcity, limiting integrated service provision: Shortages of essential materials and equipment required for providing integrated CCS services within ART clinics directly impede the ability of healthcare providers to offer comprehensive care in a single visit. This scarcity necessitates separate appointments and potentially longer waiting times for patients, undermining the very efficiencies that integration could offer.

*“There are challenges in the integration of HIV and CCS service for HIV-positive women: primarily, the government has no specific policies of integration in the ground. Lack of materials which are important for service delivery, the HFs have no adequate and apparent dark classrooms and, the government not well understood the burden of this disease [cervical cancer] on mothers, especially WLHIV.”* (38 years, IDKII-6).

This quote underscores the importance of binding policies to standardize the delivery of integrated CCS and ART services and illustrates the practical challenges posed by material shortages.

### Theme-II: health institution limitations impeding integrated service delivery

This theme highlights key infrastructural and human resource constraints within health institutions that significantly hinder the effective integration of CCS services into HIV care at ART follow-up clinics, ultimately impacting the scope and quality of care provided.

#### Sub-theme 1: inadequate infrastructure, restricting service integration

The shortage of suitable infrastructure, including dedicated spaces and necessary equipment within ART clinics, poses a significant barrier to offering integrated CCS services. This lack of physical resources limits the ability to conduct screenings efficiently and comfortably during ART visits, potentially leading to logistical challenges, longer wait times, and a reduced capacity to serve all eligible WLHIV. This idea was explored by one participant:

*“The barriers affecting integrated service* var*y from one health facility to another. However, the main challenges in our facility were a lack of suitable classes for the two services and, a lack of dually trained HCPs. In addition, lack of motivation and commitment to health care providers and health facility leaders.”* (35 years, in FGD-2).

#### Sub-theme 2: limited human capacity constraining integrated service provision

The insufficient number of healthcare providers adequately trained in both HIV care and cervical cancer screening within health institutions directly restricts the availability of integrated services. This human resource limitation means that even if physical space were available, there might not be enough personnel to provide both services concurrently, forcing WLHIV to make separate visits and potentially miss out on crucial preventive care.

In the same way, this idea was also stated by another respondent in another health institution:

*“The main challenge in our institution is the lack of adequate classrooms favorable for both CC screening and ART service as well as not well understood the burden of CC on mothers. (…) both the institution itself and health care providers have no motivation to integrate the two services are main barriers to our institution.”* (34 years, IDKII-7).

### Theme-III: clinician-related factors impeding integrated service uptake

This theme highlights key issues related to healthcare providers that act as significant impediments to the successful integration of CCS within ART follow-up clinics, ultimately affecting the willingness and ability of clinicians to deliver these combined services effectively.

#### Sub-theme 1: lack of motivation and commitment hindering integrated service delivery

Insufficient motivation and commitment among some healthcare providers toward adopting new integrated service models can lead to a reluctance to prioritize and actively participate in their implementation. This lack of engagement can result in inconsistent service delivery, underutilization of integrated protocols, and a potential failure to fully realize the benefits of co-located care for WLHIV. Participants highlighted that integrating these services, being a novel approach, demands strong motivation and commitment from both HCPs and HFLs. It stated as.

*“In my understanding, the main challenges are health care providers have no interest in performing this activity, especially those who work at ART, they reasoning an overlapping of activity, there is no adequately trained personnel, and have no outpatient department (OPD) for CC screening and it is not performed as independent jobs.”* (36 years, in FGD-2).

#### Sub-theme 2: insufficient skills and experience, limiting integrated service provision

Gaps in the necessary skills and practical experience among healthcare providers in delivering both CCS and ART services create a significant barrier to effective integration. This lack of dual expertise can lead to hesitancy in offering both services, the potential for errors or suboptimal care, and the need for separate referrals, thereby undermining the goal of streamlined, comprehensive care within a single visit.

Furthermore, they noted a significant shortage of personnel trained and experienced in delivering both ART and CCS services concurrently. One respondent illustrated this point: Regarding this idea, another respondent also stated:

*“As our organization, in addition to lack of suitable classroom and trained and skilled personnel, they also have no motivation and initiation to integrate these services [HIV care and cervical cancer screening] as (new initiative) in addition, no one well understood the burden of this problem [Cervical cancer] on mothers, particularly in these high-risk women.”* (37 years, IDKII-4).

### Theme IV: leveraging existing resources for enhanced service integration

This theme underscores how the pre-existing availability of key resources within the health system can be strategically leveraged to facilitate the successful integration of ART and CCS services for WLHIV, ultimately improving service delivery and efficiency.

#### Sub-theme 1: accessible vertical programs as a foundation for integration

The established and widespread availability of both free ART and CCS services as independent vertical programs provides a crucial existing infrastructure and patient base upon which integrated services can be built. This pre-existence eliminates the need to create services from scratch, allowing for a more streamlined integration process by merging existing pathways and potentially reaching a larger population of WLHIV. Participants frequently expressed these ideas, as follows:

*“(…*) *In my understanding, the integration of CCS is possible, because, the mother can get ART service by assigned trained HCP, and in the same way, CCS service at gynaecologic (OPD) by midwives, so that these services that have been delivered at separate OPD can be given as integrated service by the same HCPs at the same place simultaneously.”* (35 years, in FGD-1).

#### Sub-theme 2: cross-trained healthcare providers enabling multipurpose service delivery

The presence of HCPs already trained in either ART or CCS represents a valuable human resource that can be further leveraged through cross-training or task-shifting to deliver integrated services within a single point of care. This adaptability of the workforce allows for the efficient utilization of existing personnel, potentially reducing the need for additional specialized staff and enabling the provision of comprehensive care by the same provider. One participant stated:

*“In our institution, the screening method is VIA, it is so simple, that can be performed by trained internal staff, so, this activity [integration] cannot require additional external staff for this procedure, if trained any HCP can do the procedure, either the HCP at ART can do CCS or who trained by CCS can do the ART service after training. Otherwise, integration service can be delivered by assigned HCP in the two separate units at the same place.”* (37 years, in FGD-2).

#### Sub-theme 3: VIA method’s simplicity facilitating integration within existing clinics

The ease of use and the ability of existing clinic staff to perform the VIA method for cervical cancer screening make it a highly compatible component for integration within ART follow-up clinics. Its simplicity minimizes the need for highly specialized personnel or complex equipment, allowing for seamless incorporation into routine ART visits and expanding access to essential screening without significant disruption to existing workflows:

Likewise, another participant also stated this idea as follows:

*“The available HCPs working at gynaecologic (OPD), can perform the screening, since the VIA method is not complex, it is a good opportunity for providing this [screening] service. Furthermore, HCPs could give the service with simple training.”* (37 years, in FGD-1).

#### Sub-theme 4: perceived benefits of service integration

Participants emphasized the critical importance of integrating CCS with ART clinics for WLHIV. This integration was seen as essential to protect patient confidentiality and prevent information leaks, which are major concerns due to the stigma and discrimination WLHIV often face. When CCS is conducted in separate, general gynecology outpatient departments (OPDs), there is a heightened risk of sensitive health information being shared with uninvolved individuals. Participants articulated this concern as follows:

*“Yes, it is important as it [integration] decreases suffering of women by being here and there at standalone clinics for HIV care and cervical cancer screening separately, increase satisfaction on health care providers, furthermore, it saves transport cos for the mothers and time for the mothers as well for healthcare providers.”* (35 years, IDKII-3).

*“The integration of these services [ART and CCS services] has paramount importance for WLHIV: First it can decrease the follow-up day, thereby reducing transport cost; secondly it decreases women’s waiting time for both services [ART and CCS service] by providing the two services at the same place [health care unit] for WLHIV. In addition, it can prevent leakage of health information for uninvolved people.”* (38 years, in FGD-3).

*“It [integrated screening] is important, for them [women] it has many advantages; when they screened at the same place by the same healthcare provider, they are not embarrassed, it helps not to disclose their secret, it saves time and decreases transport cost by decreasing follow-up days, it decreases their fear when seen [screen] by a healthcare provider familiar with them due to this many women will be screened.”* (37 years, in FGD-2).

Participants expressed this sentiment as follows:

*“Yes (…) screening the mother after reaching the advanced stage of cervical cancer and early detection are different, screen them [women] as early as before reaching the advanced stage gives mental satisfaction for health care providers as well as decrease embarrassment among them due to complicated characteristics of invasive stage of CC.”* (31 years, IDKII-2).

## Discussion

This study investigated the barriers and facilitators of integrating CCS and preventive treatment within ART follow-up clinics for WLHIV. Our key findings reveal a complex interplay where infrastructural deficits, particularly the lack of dedicated space, and a critical shortage of dually trained HCPs significantly impede integration. This echoes infrastructure and human resource limitations reported in qualitative studies in Uganda (31) and broader systematic review literature (29, 39), underscoring the systemic challenges in resource-limited settings.

Furthermore, clinician-related factors such as low motivation and insufficient skills in both CCS and ART present substantial barriers. This aligns with qualitative research in low- and middle-income countries (40), suggesting that the increased workload and coordination demands of integrated care can affect provider buy-in. While integrated care is a promising approach requiring coordinated health systems and engaged HCPs (41), our findings highlight that resource constraints and, crucially, low provider motivation are major obstacles to successful implementation, consistent with qualitative studies across SSA (31, 36, 42). This suggests a need for targeted interventions to address provider workload, enhance training, and improve motivation to foster the adoption of integrated models.

The absence of clear national integration policies and limited robust evidence on the cost-effectiveness and clinical outcomes of integrated services further hinders progress. This is consistent with qualitative studies in Malawi and Kenya (30, 43) and reinforced by a systematic review (29). This indicates that the lack of definitive data impedes evidence-based policymaking and resource allocation. Compounding these issues are funding gaps and inadequate logistical support, as also reported in a qualitative study in Ethiopia (44). These policy and resource limitations underscore the urgent need for research evaluating the impact of integrated services and the development of supportive national guidelines.

Despite these challenges, existing ART and CCS programs provide a crucial foundation for integration. Their co-location and accessibility within facilities can streamline the process, as supported by findings from qualitative studies in Zimbabwe (37). The feasibility of VIA by existing staff and the presence of HCPs trained in either ART or CCS offer opportunities for efficient cross-training. Integrated services can also foster stronger provider-patient relationships, potentially increasing trust and screening uptake, as seen from a qualitative study in Texas (45). Leveraging existing program infrastructure and focusing on cross-training initiatives are key strategies for successful integration.

Participants also perceived integrated services as potentially cost-saving, aligning with Kenyan evidence (46). Importantly, integration preserves patient confidentiality and reduces the stigma associated with dual diagnoses, a concern in Ugandan studies (31, 47). This holistic approach can minimize costs, reduce patient burden, and facilitate early detection and treatment, consistent with findings from Kenya and Zimbabwe (48, 49), potentially contributing to reduced maternal mortality. These perceived benefits and the potential for improved patient outcomes and reduced stigma provide a strong rationale for pursuing integrated care models.

## Strengths of the study

This study demonstrates several notable strengths. First, the utilization of both FGDs and IdKIIs facilitated the collection of rich and nuanced data. This dual-method approach allowed for a more comprehensive understanding of the barriers and facilitators to service integration by capturing both collective perspectives and individual insights. Second, methodological rigor was ensured through data triangulation across diverse health professionals directly involved in ART and CCS (Chronic Care Services), as well as HFLs. This triangulation was further strengthened by the use of audio recordings and detailed field notes. Furthermore, the study enhanced the trustworthiness of its findings through member checking, engaging participants to validate interpretations, and peer debriefing with disinterested colleagues before finalizing the results.

## Limitations of the study

The findings of this study should be interpreted with its limitations in mind. First, the focus on nine purposefully selected HFs within the Awi Zone introduces potential selection bias, as these sites may not represent all ART centers, limiting the generalizability of findings. Second, the restriction to one zone in Northwest Ethiopia may limit the transferability of these insights to other regions with different healthcare contexts. Therefore, the findings offer a context-specific understanding that may not be directly applicable to all settings. Furthermore, the lack of policymakers participating in this study may hindered some views and perspectives, which might limitation in representativeness.

## Conclusion and recommendations

This qualitative study revealed that integrating CCS into HIV care at ART clinics for WLHIV is hindered by significant barriers. Institutional limitations, including infrastructure deficit and shortages of dually trained HCPs, were primary obstacles. Health system deficiencies, such as the absence of clear integration policies and funding, and healthcare provider challenges, notably low motivation and skill gaps, further compounded these issues. Conversely, existing vertical ART and CCS programs, the simplicity of VIA screening, and the potential for enhanced patient confidentiality emerged as key facilitators. The integration of these services is crucial, streamlining care, safeguarding privacy, and enabling timely cancer detection and treatment for WLHIV.

Thus, to optimize the integration of CCS and treatment within HIV care, a comprehensive strategy is required. Health institutions should establish dedicated spaces for integrated services and invest in targeted training to cultivate a workforce proficient in both ART and CCS. Concurrently, strengthening health system frameworks is essential. These include formulating and implementing clear national policies supporting integrated care, generating evidence on cost-effectiveness, and clinical outcomes, and securing sustainable funding mechanisms. Crucially, enhancing HCP engagement through motivational strategies and training programs is vital for improving commitment and skill levels. Furthermore, future research and implementation efforts should focus on longitudinal studies to assess long-term impacts and cost-effectiveness evaluations of integration models.

## Data Availability

The raw data supporting the conclusions of this article will be made available by the authors, without undue reservation.

## References

[1] Center for Desease Control and Preventions. Inside knowledge about gynecologic cancer. Factsheet. (2018). Available onoline at: https://www.cdc.gov/cancer/cervical/pdf/cervical_facts.pdf (Accessed November 26, 2024).

[2] WHO. (2020). Cervical cancer elimination initiative: from call to action for a global movement. Available online at: https://cdn.who.int/media/docs/default-source/cervical-cancer/230519-ccei-brochure.pdf (Accessed December 24, 2024.

[3] Mayo Clinic Press. Overview of cervical cancer, syptom and causes. Available online at: https://www.mayoclinic.org/diseases-conditions/cervical-cancer/symptoms-causes/syc-20352501 (Accessed August 17, 2024.

[4] National Cervical Cancer Coalition. Stages of cervical cancer. American sexual health association (2022). Available online at: https://www.nccc-online.org/hpvcervical-cancer/stages-of-cervical-cancer/ (Accessed September 25, 2024).

[5] World Health Organization, Cervical cancer overview. Available online at: https://www.who.int/news-room/fact-sheets/detail/cervical-cancer?gad_source=1&gclid=CjwKCAiA65m7BhAwEiwAAgu4JErUmwZCioOAFAvDZy9lY6l47S9VvXsqc6CNaONBwRE1svd9I_fe0xoCpJAQAvD_BwE (Accessed December 21, 2024.

[6] European society for medical oncology. (2022). First blobal estimates of cervical cancer attributable to HIV. Available online at: https://www.esmo.org/oncology-news/first-global-estimates-of-cervical-cancer-attributable-to-hiv (Accessed August 15, 2024.

[7] UNAIDS. (2019). The little-known links between cervical cancer and HIV. Available online at: https://www.unaids.org/en/resources/presscentre/featurestories/2019/may/20190531_cervical-cancer-hiv (Accessed December 20, 2024).

[8] Stelzle D Tanaka LF Lee KK Khalil AI Baussano I Shah AS. Estimates of the global burden of cervical cancer associated with HIV. Lancet Glob Health. (2021) 9:e161–9. doi: 10.1016/S2214-109X(20)30459-9 33212031 PMC7815633

[9] WHO. WHO releases new estimates of the global burden of cervical cancer associated with HIV [202]. Available online at: https://www.who.int/news/item/16-11-2020-who-releases-new-estimates-of-the-global-burden-of-cervical-cancer-associated-with-hiv (Accessed July 21, 2024).

[10] Castle PE Einstein MH Sahasrabuddhe VV. Cervical cancer prevention and control in women living with human immunodeficiency virus. CA Cancer J Clin. (2021) 71:505–26. doi: 10.3322/caac.21696, PMID: 34499351 PMC10054840

[11] Ibrahim Khalil A Mpunga T Wei F Baussano I de Martel C Bray F. Age-specific burden of cervical cancer associated with HIV: a global analysis with a focus on sub-Saharan Africa. Int J Cancer. (2022) 150:761–72. doi: 10.1002/ijc.33841, PMID: 34626498 PMC8732304

[12] WHO. Global strategy to accelerate the elimination of cervical cancer as a public health problem (2020). Available online at: https://iris.who.int/bitstream/handle/10665/336583/9789240014107-eng.pdf (Accessed Septenber 16, 2024).

[13] Rihana N Nanjappa S Sullivan C Velez AP Tienchai N Greene JN. Malignancy trends in HIV-infected patients over the past 10 years in a single-center retrospective observational study in the United States. Cancer Control. (2018) 25:1073274818797955. doi: 10.1177/1073274818797955, PMID: 30185062 PMC6128080

[14] Rohner E Bütikofer L Schmidlin K Sengayi M Maskew M Giddy J. Cervical cancer risk in women living with HIV across four continents: a multicohort study. Int J Cancer. (2020) 146:601–9. doi: 10.1002/ijc.32260, PMID: 31215037 PMC6898726

[15] HIV infection and malignancy: management considerations (2023). Available online at: https://tkl.uptodate.com/contents/hiv-infection-and-malignancy-management-considerations/print (Accessed November 10, 2024).

[16] Denslow SA Rositch AF Firnhaber C Ting J Smith JS. Incidence and progression of cervical lesions in women with HIV: a systematic global review. Int J STD AIDS. (2014) 25:163–77. doi: 10.1177/0956462413491735, PMID: 24216030 PMC5524184

[17] D’andrea F Pellicanò G Venanzi Rullo E d’Aleo F Facciolà A Micali C. Cervical cancer in women living with HIV: a review of the literature. World Cancer Res J. (2019) 6:e1224.

[18] Dessalegn Mekonnen B. Cervical cancer screening uptake and associated factors among HIV-positive women in Ethiopia: a systematic review and meta-analysis. Adv Prev Med. (2020) 2020:7071925. doi: 10.1155/2020/7071925, PMID: 32879739 PMC7448202

[19] Ayenew AA Zewdu BF Nigussie AA. Uptake of cervical cancer screening service and associated factors among age-eligible women in Ethiopia: systematic review and meta-analysis. Infect Agents Cancer. (2020) 15:1–17. doi: 10.1186/s13027-020-00334-3, PMID: 33292388 PMC7666476

[20] Shah R Nwankwo C Kwon Y Corman SL. Economic and humanistic burden of cervical cancer in the United States: results from a nationally representative survey. J Women's Health. (2020) 29:799–805. doi: 10.1089/jwh.2019.7858, PMID: 31967943 PMC7307680

[21] Dryden-Peterson S Bvochora-Nsingo M Suneja G Efstathiou JA Grover S Chiyapo S. HIV infection and survival among women with cervical cancer. J Clin Oncol. (2016) 34:3749–57. doi: 10.1200/JCO.2016.67.9613, PMID: 27573661 PMC5477924

[22] Salihu H Dongarwar D Ikedionwu CA Shelton A Jenkins CM Onyenaka C. Racial/ethnic disparities in the burden of HIV/cervical cancer comorbidity and related in-hospital mortality in the USA. J Racial Ethn Health Disparities. (2021) 8:24–32. doi: 10.1007/s40615-020-00751-5, PMID: 32378158

[23] Endale H Mulugeta T Habte T. The socioeconomic impact of cervical cancer on patients in Ethiopia: evidence from tikur anbessa specialized hospital. Cancer Manag Res. (2022):1615–25. doi: 10.2147/CMAR.S352389 35535268 PMC9078746

[24] Fauk NK Mwanri L Hawke K Mohammadi L Ward PR. Psychological and social impact of HIV on women living with HIV and their families in low-and middle-income Asian countries: A systematic search and critical review. Int J Environ Res Public Health. (2022) 19:6668. 35682255 10.3390/ijerph19116668 PMC9180788

[25] Oduguwa E Dongarwar D Salihu HM. Trends in premature deaths among women living with HIV/AIDS and cervical Cancer. South Med J. (2020) 113:651–8. doi: 10.14423/smj.0000000000001184, PMID: 33263137

[26] Shoja Z Farahmand M Hosseini N Jalilvand S. A meta-analysis on human papillomavirus type distribution among women with cervical neoplasia in the WHO eastern mediterranean region. Intervirology. (2019) 62:101–11. doi: 10.1159/000502824, PMID: 31527382

[27] WHO, New World health organization’s recommendations on the screening and treatment to prevent cervical cancer among women living with HIV (2021). Available onoine at: https://www.who.int/publications/i/item/9789240030961 (Accessed Nevember 19, 2024).

[28] Odafe S Torpey K Khamofu H Oladele E Adedokun O Chabikuli O. Integrating cervical cancer screening with HIV care in a district hospital in Abuja, Nigeria. Niger Med J. (2013) 54:176–84. doi: 10.4103/0300-1652.114590, PMID: 23901180 PMC3719244

[29] Sigfrid L Murphy G Haldane V Chuah FLH Ong SE Cervero-Liceras F. Integrating cervical cancer with HIV healthcare services: a systematic review. PLoS One. (2017) 12:e0181156. doi: 10.1371/journal.pone.0181156, PMID: 28732037 PMC5521786

[30] Vodicka EL Babigumira JB Mann MR Kosgei RJ Lee F Mugo NR. Costs of integrating cervical cancer screening at an HIV clinic in Kenya. Int J Gynecol Obstet. (2017) 136:220–8. doi: 10.1002/ijgo.12025, PMID: 28099724

[31] Kumakech E Andersson S Wabinga H Berggren V. Integration of HIV and cervical cancer screening perceptions and preferences of communities in Uganda. BMC Womens Health. (2015) 15:1–13. doi: 10.1186/s12905-015-0183-4, PMID: 25783655 PMC4359479

[32] Watt N Sigfrid L Legido-Quigley H Hogarth S Maimaris W Otero-García L. Health systems facilitators and barriers to the integration of HIV and chronic disease services: a systematic review. Health Policy Plan. (2017) 32:iv13. doi: 10.1093/heapol/czw149, PMID: 28666336 PMC5886067

[33] FMOH. (2015). Guideline for cervical cancer prevention and control in Ethiopia. Available onoine at: https://www.iccp-portal.org/system/files/plans/Guideline%20Eth%20Final.pdf

[34] Ethiopia Federal Minstry of Health. (2022). National guidelines for comprehensive HIV prevention, care and treatment. Available online at: https://www.afro.who.int/publications/national-consolidated-guidelines-comprehensive-hiv-prevention-care-and-treatment (Accessed September 23, 2024).

[35] Awi Zone Heealth Department (2022) Annual report of Awi Zone health department

[36] Randall TC Ghebre R. Challenges in prevention and care delivery for women with cervical cancer in sub-Saharan Africa. Front Oncol. (2016) 6:160. doi: 10.3389/fonc.2016.00160, PMID: 27446806 PMC4923066

[37] Sibanda E Ruhode N Madanhire C Hatzold K Cowan F. Barriers and facilitators to uptake of cervical cancer screening among clients attending integrated HIV/sexual and reproductive health clinics in Zimbabwe. BMJ Publishing Group Ltd. (2015).

[38] Auma J. Exploring effective approaches of integrating cervical screening services into the normal routine care within HIV clinics: a qualitative study in a Ugandan community health facility. (United Kingdom: Master’s thesis, University of Salford). (2021).

[39] Auschra C. Barriers to the integration of care in inter-organisational settings: a literature review. Int J Integr Care. (2018) 18:5. doi: 10.5334/ijic.3068, PMID: 29632455 PMC5887071

[40] Kassa RN Shifti DM Alemu K Omigbodun AO. Integration of cervical cancer screening into healthcare facilities in low-and middle-income countries: a scoping review. PLoS Glob Public Health. (2024) 4:e0003183. doi: 10.1371/journal.pgph.0003183, PMID: 38743652 PMC11093339

[41] Belhadj H Rasanathan JJK Denny L Broutet N. Sexual and reproductive health and HIV services: integrating HIV/AIDS and cervical cancer prevention and control. Int J Gynecol Obstet. (2013) 121:S29–34. doi: 10.1016/j.ijgo.2013.02.002, PMID: 23477703

[42] Lott BE Halkiyo A Kassa DW Kebede T Dedefo A Ehiri J. Health workers’ perspectives on barriers and facilitators to implementing a new national cervical cancer screening program in Ethiopia. BMC Womens Health. (2021) 21:185. doi: 10.1186/s12905-021-01331-3, PMID: 33941159 PMC8090515

[43] Mwagomba BLM Matanje Mwagomba BL Ameh S Bongomin P Juma PA MacKenzie RK. Opportunities and challenges for evidence-informed HIV-noncommunicable disease integrated care policies and programs: lessons from Malawi, South Africa, Swaziland and Kenya. AIDS. (2018) 32:S21–32. doi: 10.1097/QAD.0000000000001885, PMID: 29952787

[44] Badacho AS Mahomed OH. Facilitators and barriers to integration of noncommunicable diseases with HIV care at primary health care in Ethiopia: a qualitative analysis using CFIR. Front Public Health. (2023) 11:1247121. doi: 10.3389/fpubh.2023.1247121, PMID: 38145060 PMC10748758

[45] Fletcher FE Buchberg M Schover LR Basen-Engquist K Kempf MC Arduino RC. Perceptions of barriers and facilitators to cervical cancer screening among low-income, HIV-infected women from an integrated HIV clinic. AIDS Care. (2014) 26:1229–35. doi: 10.1080/09540121.2014.894617, PMID: 24635664 PMC4087052

[46] Vodicka EL Chung MH Zimmermann MR Kosgei RJ Lee F Mugo NR. Estimating the costs of HIV clinic integrated versus non-integrated treatment of pre-cancerous cervical lesions and costs of cervical cancer treatment in Kenya. PLoS One. (2019) 14:e0217331. doi: 10.1371/journal.pone.0217331, PMID: 31170193 PMC6553698

[47] Ninsiima M Nyabigambo A Kagaayi J. Acceptability of integration of cervical cancer screening into routine HIV care, associated factors and perceptions among HIV-infected women: a mixed methods study at Mbarara regional referral hospital, Uganda. BMC Health Serv Res. (2023) 23:333. doi: 10.1186/s12913-023-09326-6, PMID: 37013535 PMC10071680

[48] Sibanda E Ruhode N Madanhire C Hatzold K Cowan FM. P17. 15 barriers and facilitators to uptake of cervical cancer screening among clients attending integrated hiv/sexual and reproductive health clinics in Zimbabwe. BMJ. (2015) 91:A228.3–A229. doi: 10.1136/sextrans-2015-052270.593

[49] Njuguna DK. The role of health services integration in healthcare system performance: a case of HIV/AIDS and NCD services in Nakuru County, Kenya KeMU (2024).

